# Effects of a New Patient Safety-Driven Oxytocin Dosing Protocol on Postpartum Hemorrhage

**DOI:** 10.1155/2014/157625

**Published:** 2014-04-27

**Authors:** David S. McKenna, Kari Rudinsky, Jiri Sonek

**Affiliations:** Maternal-Fetal Medicine, Department of Obstetrics and Gynecology, Miami Valley Hospital, Dayton, OH 45409, USA

## Abstract

*Objective.* To determine if there was an increase in postpartum (PP) hemorrhage after decreasing the PP oxytocin dose from 40 to 30 units. * Study Design.* Retrospective cohort study comparing 8 months before to 8 months after the change. PP day 1 hemoglobin was subtracted from admission hemoglobin. Mean change was compared by Student's *t*-test. The best fit polynomial was analyzed for trends between the two time frames. Women who received blood transfusions were excluded. * Results. *73/3564 (2.0%) women received blood transfusions in the pre group and 64/3295 (1.9%) women in the post group, *P* = 0.8. Mean hemoglobin change ± standard deviation was 1.53 ± 0.03 g/dL for pre versus 1.52 ± 0.05 g/dL for post, *P* = 0.68. 1003/3114 (32.2%) in the pre group had a hemoglobin decrease of ≥2 g/dL compared to 918/2895 (31.7%) in the post group, *P* = 0.7. 261/3114 (8.4%) in the pre group had a hemoglobin decrease of ≥3 g/dL compared to 252/2895 (8.7%), *P* = 0.7. There were no significant trends between the two time frames. * Conclusion.* The change in the dose of PP oxytocin did not result in an increase in postpartum hemorrhage or an increase in the need for blood transfusion.

## 1. Background

In their landmark report, the Institute of Medicine noted that errors in health care are a significant cause of death and injury [1]. In response to the national interest to reduce health care errors, the American College of Obstetricians and Gynecologists recommended the implementation of medication practices to improve patient safety [2]. When implementing change for improvement, there is always the potential for adverse secondary effects, known as balancing measures [3]. The Hospital Corporation of America (HCA) has incorporated many patient safety initiatives including a conservative standardized oxytocin dosing regimen, part of which is a standard concentration of 15 units oxytocin per liter of intravenous fluid [4]. Clark et al. reported improved maternal and newborn outcomes with the implementation of the HCA's oxytocin protocol [5]. Subsequently, many hospitals including ours adopted a similar protocol in the interest of improving patient outcomes [6, 7].

In the past our labor and delivery unit had prepared oxytocin at a concentration of 20 units/L. With this concentration, the displayed rate on the infusion pump did not numerically match the dose of oxytocin that was being infused. For every 3.0 mL/hr of infusion, only 1.0 milliunit of oxytocin was infused per minute. This was thought to be ambiguous and a potential source for medication errors. Therefore our unit changed the oxytocin concentration as part of the HCA's standardized oxytocin dosing protocol. The concentration of oxytocin was changed to 30 units of oxytocin per 500 mL of intravenous fluid. The concentration was standardized so that the infusion rate was numerically equivalent to the oxytocin delivery rate (i.e., 1.0 mL/hr = 1.0 milliunit/min). As a result, the standardized dosing related to postpartum oxytocin administration also changed. In the past a 1 liter infusion of normal saline with 40 units of oxytocin was given postpartum, and this was changed to a postpartum 500 mL infusion of normal saline containing 30 units of oxytocin.

Shortly after the change, obstetricians began to verbally report a perceived increase in postpartum hemorrhage. This was attributed to the decrease postpartum oxytocin dose from 40 units to 30 units. The subjective impression of the obstetricians was that a postpartum dose of 30 units of oxytocin was insufficient and was causing an increase in the rate of postpartum hemorrhage. The purpose of this study was to evaluate a balancing measure of a quality improvement initiative to standardize the dosing of oxytocin; namely, whether there was an unanticipated consequence of an increase in postpartum hemorrhage.

## 2. Methods

This is a retrospective, cohort study comparing the eight months preceding the change in medication protocol to eight months following the change in protocol. The study protocol was approved by the Miami Valley Hospital Institutional Review Board. As the study was of existing data and there was little or no risk for compromise of protected health care information, the requirement for written performed consent was waived. A search for the electronic medical record was performed in order to extract the pre and posthemoglobin values of all deliveries for the months of February 2009 until September 2009 “pre” and November 2009 through June 2010 “post,” omitting the month between these selected time frames to allow for education and adjustment to the protocol. All singleton and multiple gestation admissions resulting in vaginal or cesarean delivery were included. In addition, all patients who had a vaginal or cesarean delivery and received blood transfusions during admission were identified.

In the setting of a vaginal delivery, standard orders after the delivery of the placenta included a dose of 40 units of oxytocin in 1000 mL of lactated Ringers (LR) prior to the change and 30 units of oxytocin in 500 mL of LR after the change. The oxytocin infusion was administered at 333 cc per hour for the duration of the third stage of labor and then reduced to 150 cc/hr for the remainder of the infusion. Women undergoing a cesarean delivery received the same amount of oxytocin (either 40 units in 1000 mL LR or 30 units in 500 mL LR) which was begun in the operating room at 333 cc/hr and then completed in the recovery room at 150 cc/hr. Total time for oxytocin for both vaginal and cesarean deliveries was 2-3 hours. Per anesthesia protocol, women undergoing a cesarean delivery received an additional 20 units of oxytocin in the operating room after delivery of the placenta. The practice of administering an extra 20 units of oxytocin in the operating room did not change between the two-time periods. Women with postpartum hemorrhage received additional doses of oxytocin and other uterotonic agents including methylergonovine, prostaglandin F2 alpha, and prostaglandin E1, per the obstetrician's discretion.

Per preset admission orders on the electronic medical record (EMR), all inpatient deliveries obtain a complete blood count (CBC) on admission and a CBC on postpartum day 1 (PPD 1). The time that elapsed between the CBC obtained on admission and the PPD 1 CBC varied from 12 to 24 hours. Women who received a blood transfusion or who lacked an admission or PPD 1 hemoglobin were excluded.

GraphPad Prism (GraphPad Software, San Diego, CA) was used for statistical analyses. The mean change in hemoglobin was compared between the two groups by Student's *t*-test. The mean change in hemoglobin was calculated for each month and a best fit multiple regression polynomial curve was constructed for each time series and compared by the extra sum of squares *F* test. Women who received blood products were excluded from the hemoglobin analyses. The rates of blood transfusion in the two groups were compared using Fisher's exact test. A *P* value less than 0.05 was considered statistically significant.

## 3. Results 

There were 3564 deliveries in the pregroup, and 3295 deliveries in the postgroup. There were 73/3564 (2.0%) women who received blood transfusions in the pregroup and 64/3295 (1.9%) women in the postgroup, *P* = 0.8. The women who received transfusions were excluded from the subsequent analyses. There was incomplete hemoglobin data on 377 deliveries (10.6%) in the pregroup and 336 (10.2%) in the post group, leaving 3114 and 2895 deliveries, respectively, for the hemoglobin analyses. The cesarean delivery rate was 31.1% in the pregroup and 30.0 in the postgroup.

**Figure 1:**
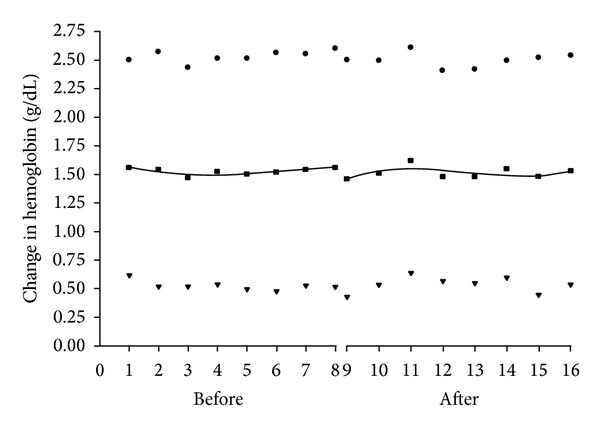
Mean hemoglobin (g/dL) change (squares) pre (months 1–8) and post (months 9–16). Two standard deviations above (circles) and below (triangles). The curve for the mean hemoglobin is the best fit third-degree polynomial.

Figure 1 graphically displays the monthly mean changes in hemoglobin over the two periods. The mean change ± standard deviation in pre to postdelivery hemoglobin was 1.53 ± 0.03 g/dL for the pregroup versus 1.52 ± 0.05 g/dL for the postgroup 2, *P* = 0.68. The trend over both eight-month periods was best modeled with a third-degree polynomial, and the curves were not statistically distinguishable, *P* = 0.34.

**Figure 2:**
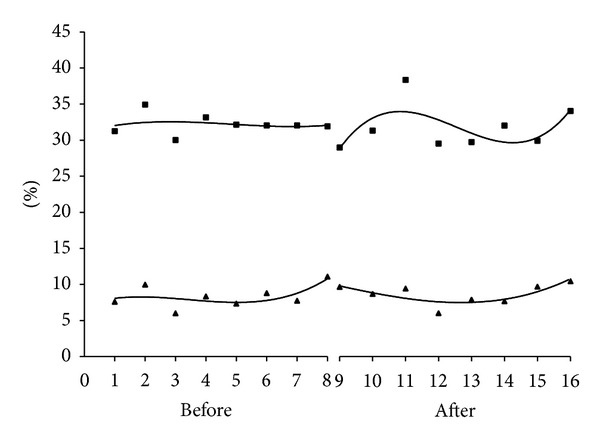
Percent of deliveries with a change in hemoglobin greater than 3 g/dL (triangles) and greater than 2 g/dL (squares). Pre (months 1–8) and post (months 9–16). Curves are best fit third-degree polynomial.

There were 1003/3114 (32.2%) women in the pregroup with a hemoglobin decrease of at least 2 g/dL, compared to 918/2895 (31.7%) in the postgroup, *P* = 0.7. There were 261/3114 (8.4%) women in the pregroup with a hemoglobin decrease of 3 g/dL or greater, compared to 252/2895 (8.7%), *P* = 0.7.  Figure 2 displays the number of women per month who had a change in hemoglobin exceeding 2 g/dL and 3 g/dL. The series were modeled by a third-degree polynomial, and there was not a significant difference between pre and post for a hemoglobin change of 2 g/dL (*P* = 0.44) nor 3 g/dL (*P* = 0.21).

## 4. Conclusion

In this study we evaluated a standard postpartum 30-unit oxytocin dose versus a 40-unit dose and found there was no difference in the need for blood transfusion, mean hemoglobin change, and hemoglobin changes of 2 or 3 g/dL, demonstrating that the new medication protocol did not increase the incidence of postpartum blood loss nor number of blood transfusions despite administering a lower total dose of oxytocin. The effects of medical errors are far reaching and costly and have the potential for high liability, especially when they occur on the labor and delivery unit [8]. Standardized medication dosing may have a positive impact on the reduction of medical errors. However it should be emphasized that while standardization can have medical advantages, there may also be unanticipated disadvantages, that is, balancing measures.

In the United States the prophylactic use of oxytocin during the third stage of labor for the prevention of uterine atony and postpartum hemorrhage is the accepted practice [9]. Despite this wide spread practice there is insufficient data and little agreement or evidence to recommend an optimal dose of oxytocin and 10 to 40 units are usually given [10, 11]. Recently, Tita et al. compared three different third-stage oxytocin doses (80 units, 40 units, or 10 units) and did not find a difference in the incidence of uterine atony or postpartum hemorrhage [12]. However the 80-unit dose was found to reduce the need for treatment of hemorrhage after the first postpartum hour and fewer women had a decline of hematocrit of 6% or more [12].

The strength of this study lies in its large sample size, as outcomes from approximately 6000 deliveries were included in analysis. In addition, the use of the electronic medical record allowed for accuracy in assessment of pre and postpartum hemoglobin levels as well as rates of transfusions. This study had several limitations which are attributable to the retrospective design: (1) the subjects were not stratified by mode of delivery; however, the percentage of cesarean deliveries during the two periods were not significantly different, (2) the baseline characteristics of the sample populations were not examined in detail, and (3) general assumptions are made regarding similarities between the cohorts studied. The two cohorts were not evaluated with respect to any differences regarding the existence of maternal bleeding disorders or other factor such as antepartum or intra-partum complications, which might increase the risk of postpartum hemorrhage. The study does not take into account changes in provider practice over time, repeated administration of oxytocin in the postpartum period, nor the use of additional uterotonics. Many of these limitations could be overcome with a prospective study. The use of admission and postpartum day 1 hemoglobin values also has some limitations as it is not a direct measurement of blood loss, but its use has been reported in the literature as a surrogate to measuring actual blood loss [12–14].

We have demonstrated that standardized oxytocin dosing resulting in a total decreased amount of medication delivered in the postpartum period does not result in an increased amount of postpartum hemorrhage. While this study exemplifies one aspect of obstetric care that has not been affected by medication standardization, many more avenues for research exist. Certainly, as quality improvement moves to the forefront of hospital evaluation and performance measurements, one can expect to see more and more implementations meant to simplify procedure and minimize error. It is imperative that we remain cognizant of the potential unanticipated adverse effects that may occur with new protocols.
